# Recurrent Fevers, Dysautonomia, and Dehydration in a Patient With Lesch-Nyhan Syndrome

**DOI:** 10.7759/cureus.61170

**Published:** 2024-05-27

**Authors:** Samuel Pan, Annie Truss, Sabiha Hussain

**Affiliations:** 1 Department of Medicine, Mount Sinai Hospital, New York, USA; 2 Department of Family Medicine, Rutgers Robert Wood Johnson Medical School, Piscataway, USA; 3 Division of Pulmonary and Critical Care Medicine, Robert Wood Johnson University Hospital, New Brunswick, USA

**Keywords:** hyperthermia, dopamine signaling, neuroleptic malignant syndrome, dysautonomia, lesch-nyhan syndrome

## Abstract

Lesch-Nyhan syndrome (LNS) is a disease characterized by a reduced ability to recycle purines, leading to increased de novo purine synthesis and uric acid production. Patients classically present with an array of hyperuricemic, neurologic, and behavioral symptoms. In this report, we describe a 26-year-old male with a history of LNS and recurrent fevers of unknown origin who presented to the emergency department (ED) with a fever, hypotension, and hypernatremia. We suspect that our patient's presentation was caused by autonomic instability in the setting of LNS leading to excessive free water loss. This report highlights a rare but life-threatening manifestation of LNS.

## Introduction

Lesch-Nyhan syndrome (LNS) is an X-linked recessive genetic disorder characterized by decreased hypoxanthine-guanine phosphoribosyltransferase (HGPRT) enzyme activity, leading to impaired conversion of hypoxanthine and guanine to inosine monophosphate (IMP) and guanosine monophosphate (GMP), respectively. The subsequent buildup of hypoxanthine and guanine results in excess uric acid synthesis, manifesting clinically as gouty arthritis and nephrolithiasis. Patients also classically display dystonia, choreoathetosis, intellectual disability, and self-mutilation.

Treatment of LNS is multifactorial with no definitive cure. Hyperuricemia is treated with allopurinol, a xanthine oxidase inhibitor that inhibits the production of uric acid from hypoxanthine. Spasticity is managed symptomatically with baclofen or benzodiazepines. Behavioral symptoms are managed with stress management and soft restraints. Antipsychotics may be administered, although results have been mixed [1].

Recently, cases of paroxysmal autonomic insufficiency in LNS patients manifesting as extreme hyperthermia, hypotension, tachycardia, and tachypnea have been reported, drawing attention to this underrecognized feature of the disease [2,3]. Surprisingly, these episodes were accompanied by concurrent neuroleptic malignant syndrome (NMS)-like symptoms [2,3].

NMS is a disease usually precipitated by dopamine (DA) blockers or withdrawal of dopaminergic medications and characterized by hyperthermia, muscle rigidity, altered mental status, and autonomic instability [4]. Risk factors consist of dehydration, malnutrition, physical exhaustion, heat exposure, and psychoactive substances [5]. Treatments vary depending on presentation and include stopping the offending agent or reintroducing the dopaminergic medication, reducing body temperature with cooling blankets or ice packs, relaxing the muscles with dantrolene, and overcoming the DA blockade with bromocriptine.

The etiology of the neurologic, behavioral, and autonomic manifestations in LNS is unknown, but it is postulated that aberrant DA signaling may play a causal role [6-9]. DA is a neurotransmitter responsible for movement and thermoregulation. Dysfunction of DA pathways may account for the overlapping motor (dystonia, dysarthria, and dysphagia) and thermoregulatory (temperature tolerance and hyperthermia) deficits seen in LNS, NMS, and Parkinson's disease (PD) [10-13].

Here, we report a patient with a history of LNS and recurrent hospitalizations for non-infectious fevers presenting with a fever, dysautonomia, and severe dehydration.

## Case presentation

A 26-year-old male with a history of LNS, type 2 renal tubular acidosis, esophageal strictures, neurogenic bladder, and recurrent hospitalizations for fever presented to the emergency department (ED) with a fever and labile blood pressure.

Six years prior, the patient was hospitalized for a fever. The infectious workup was negative, and the fever self-resolved after several days. These episodes of non-infectious, self-resolving fevers ranging between 100°F and 105°F then began occurring multiple times per year.

In the emergency department, the patient was febrile to 105°F, tachycardic to 180 bpm, and tachypneic to 50 breaths per minute. His blood pressure and oxygen saturation were labile, fluctuating between 70/40 and 120/70 mmHg and 80% and 95% on room air, respectively. On physical examination, he was profusely diaphoretic, nonverbal, and wheelchair-bound. He required soft restraints due to self-mutilating behavior and demonstrated severe dystonia in his upper and lower extremities. He had a jejunostomy tube (for feeding) and gastrostomy tube (for venting) present, which both appeared normal. Initial laboratory results were concerning for an elevated lactate but otherwise largely unremarkable as shown in Table 1. Urinalysis revealed mixed gram-positive organisms. Chest X-ray was negative for consolidations. Computed tomography (CT) of the abdomen and pelvis revealed an old catheter fragment in the left pulmonary artery seen on prior CT scans and was otherwise unremarkable. Blood cultures were drawn, and a midline catheter was placed. The patient was then admitted to the medical floors and treated for presumed sepsis with 1 L of 0.9% sodium chloride solution via the jejunostomy tube, 20 mg/kg of IV vancomycin, and 3.375 g of IV piperacillin-tazobactam.

**Table 1:** Laboratory values in the emergency department Relevant laboratory findings in the emergency department on initial presentation (left) and three hours after (right). The initial magnesium level was not taken. BUN: blood urea nitrogen

Parameter	Initial	3 hours later	Normal values
Sodium	156 mEq/L	170 mEq/L	135-150 mEq/L
Potassium	4.8 mEq/L	6.5 mEq/L	3.5-5.0 mEq/L
Chloride	117 mEq/L	125 mEq/L	96-106 mEq/L
Bicarbonate	20.1 mEq/L	18.4 mEq/L	22-29 mEq/L
Anion gap	19 mmol/L	27 mmol/L	4-12 mmol/L
Creatinine	0.9 mg/dL	1.8 mg/dL	0.7-1.3 mg/dL
BUN	18 mg/dL	44 mg/dL	6-24 mg/dL
Lactate	3.9 mmol/L	4.2 mmol/L	0.5-2.2 mmol/L
Magnesium	NA	2.9 mg/dL	1.8-2.6 mg/dL

Several hours later while still in the ED, the laboratory results became concerning for acute renal failure and worsening electrolyte balance despite fluid resuscitation (Table 1). The patient was given an additional liter of 0.9% sodium chloride solution via the jejunostomy tube. The midline catheter stopped functioning, leaving the patient with only one peripheral line with tenuous access that prevented further administration of medications. The patient was transferred to the medical intensive care unit (MICU) with concerns for septic and hypovolemic shock and the necessity of central access for hydration, antibiotics, calcium gluconate, and insulin infusion.

In the intensive care unit, sodium and potassium levels returned to normal levels after a total of 2 L of dextrose 5% in water and 4 L of lactated Ringer's solution were administered. Blood and urine cultures were negative. Infectious disease was consulted and believed the patient was not septic due to the lack of an infectious source, recurrent similar presentations with negative infectious workups, and treatment with multiple recent courses of antibiotics. Vancomycin and piperacillin-tazobactam were discontinued after two and five cumulative doses, respectively. The jejunostomy tube was leaking and replaced by interventional radiology. Throughout the weeklong MICU course, the patients' metabolic derangements resolved, pressors were discontinued, and inflammatory markers returned to baseline levels. Once stabilized, the patient was transferred to the medical floor.

On the floors, the patient continued to receive fluids. The course was complicated by an episode of a fever of 101.7°F and tachycardia of 170 bpm, although the infectious workup was again negative. During this time, we increased the volume of free water administration to appropriately match the rate of free water loss. The patient received a total of approximately 7 L of dextrose 5% in 0.45% sodium chloride solution on the floors. This febrile episode self-resolved, and the patient was discharged after 12 days total. At discharge, his free water intake via the jejunostomy tube was increased to prevent further episodes of dehydration.

We propose that our patient's recurrent fevers were caused by autonomic instability due to aberrant DA signaling in the setting of LNS. The hyperthermia likely caused excessive insensible fluid losses with subsequent hypotension and metabolic derangements.

## Discussion

Dysautonomia is a rare but life-threatening manifestation of LNS. Here, we add to the existing literature on this phenomenon as described [2,3].

Robust evidence suggests that reduced DA signaling causes autonomic insufficiency in LNS. In vivo, positron emission tomography using radiolabeled tracers has revealed decreased dopa decarboxylase activity (50%-70%), DA storage (30%-40%), and DA transport (50%-70%) in the caudate and putamen of LNS patients [6,9]. Lloyd et al. [7] examined the brain tissue of postmortem LNS patients and confirmed decreased dopa decarboxylase activity (80%-96%) and decreased DA concentrations in the caudate, putamen, external pallidus, and nucleus accumbens (65%-90%). DA is an essential catecholamine in the sympathetic pathway, and LNS patients have blunted stress-induced release of norepinephrine and pressor response [14,15]. DA in the autonomic nervous system also regulates core body temperature, and deficiency of DA in the hypothalamus may lead to hyperthermia [10,11,13].

Altered signaling of other neurotransmitters, in particular norepinephrine and serotonin, have also been implicated in LNS, but the findings have been mixed and irreproducible [1,7]. Hyperuricemia alone is unlikely to cause dysautonomia because some patients display only hyperuricemia-induced gout and renal stones [16].

NMS and PD also result from a blockade in DA and manifest many of the same neurologic and autonomic features of LNS. However, unlike in NMS and PD, dopaminergic therapies are not effective treatments for LNS [17]. Breese et al. [18] showed that DA agonists have different effects on behavior when given to neonatal neuronal DA-deficient rats compared to corresponding adults. This suggests a critical time window for DA signaling development and administration of potential LNS therapies.

The cause of reduced DA signaling in LNS is unknown. Guanosine triphosphate (GTP) is a compound required for DA activation, and HGPRT deficiency leads to reduced GMP and subsequent GTP production [19,20]. Taken together, the literature suggests an axis of HGPRT deficiency, reduced GTP production, and decreased dopamine signaling, ultimately leading to dysautonomia in LNS. This mechanism may additionally contribute to the neurologic and behavioral manifestations of the disease [20].

Our patient initially presented with many NMS symptoms such as hyperthermia, tachycardia, labile blood pressure, dystonia, and dysphagia, although evaluation was difficult given that several NMS symptoms were present at baseline. Laboratory values were significant for elevated electrolyte concentrations, lactate, blood urea nitrogen (BUN), and creatinine consistent with shock secondary to hypovolemia. The infectious workup was negative, and the metabolic abnormalities resolved with fluid resuscitation alone, making sepsis an unlikely diagnosis. He was taking quetiapine, an antipsychotic that potentially causes NMS, but he had been on this medication for years and improved despite continuing the medication during admission, making NMS a less likely diagnosis. We therefore diagnosed our patient with dysautonomia central to LNS, which led to hyperthermia with NMS-like features, fluid loss, and subsequent hypovolemic shock.

Fluid-electrolyte imbalance due to dehydration are common complications of NMS. Dehydration is also a risk factor for NMS; it is possible that our patient suffered from a positive feedback loop of worsening dehydration resulting in increasing hyperthermia and subsequent further worsening of dehydration and NMS-like symptoms [5].

The initial trigger for the paroxysmal, NMS-like presentation in our patient is not clear. Of note, the mother reported that the rate of the patient's tube feeds was decreased the month prior due to increased leakage around the jejunostomy tube, potentially leading to inadequate nutrition and dehydration and increasing the risk for these episodes. Importantly, this would not explain why our patient's episodes increased in frequency at age 23 without any clear trigger. In our review, one other patient began experiencing episodes at a similar age, and two others at seven years of age [2,3].

## Conclusions

This case highlights the underrecognized feature of dysautonomia in LNS and synthesizes the existing literature on reduced dopamine signaling in LNS. LNS and NMS share a similar pathophysiology of dopamine blockade, which accounts for the difficulty in distinguishing baseline LNS features from life-threatening NMS-like episodes. We suspect that our patient's presentation was caused by autonomic instability in the setting of LNS, leading to excessive free water loss and hypovolemic shock. Differentiating infectious fevers from dysautonomic fevers is crucial for appropriate management and must be considered for LNS patients to limit the use of unnecessary antibiotics. Further, dysautonomic fevers commonly lead to severe hyperthermia and dehydration from insensible losses, making aggressive hydration and careful monitoring of electrolyte levels in these patients particularly important.
